# Does shading on great argus *Argusianus argus* feathers create a three-dimensional illusion?

**DOI:** 10.1098/rsbl.2022.0393

**Published:** 2022-11-09

**Authors:** James M. E. Firkins, Laura A. Kelley

**Affiliations:** Centre for Ecology and Conservation, University of Exeter, Penryn Campus, Penryn TR10 9FE, UK

**Keywords:** illusion, shape from shading, pictorial relief

## Abstract

Many animals use shading to infer the three-dimensional (3D) shape of objects, and mimicking natural shading patterns can produce the illusion of 3D form on a flat surface. Over 150 years ago, Charles Darwin noted that the ocelli (eyespots) on the feathers of the great argus *Argusianus argus*, when held vertically during courtship displays to females, were perfectly shaded to resemble 3D hemispheres to human viewers. We tested whether these ocelli appear 3D to birds by training chickens *Gallus gallus domesticus* to select images of either convex or concave shapes using shading cues, and then presenting them with images of great argus ocelli. Chickens successfully learned how to discriminate between convex and concave shapes, and treated the great argus pheasant ocelli in the same way as convex training stimuli. Our findings are consistent with previous studies that birds can perceive 3D shape from shading cues in a similar manner to humans. The perception of great argus ocelli as consistent with 3D shape by avian viewers suggests that shape illusions can play a role in male courtship.

## 1. Introduction

**Figure 1.**
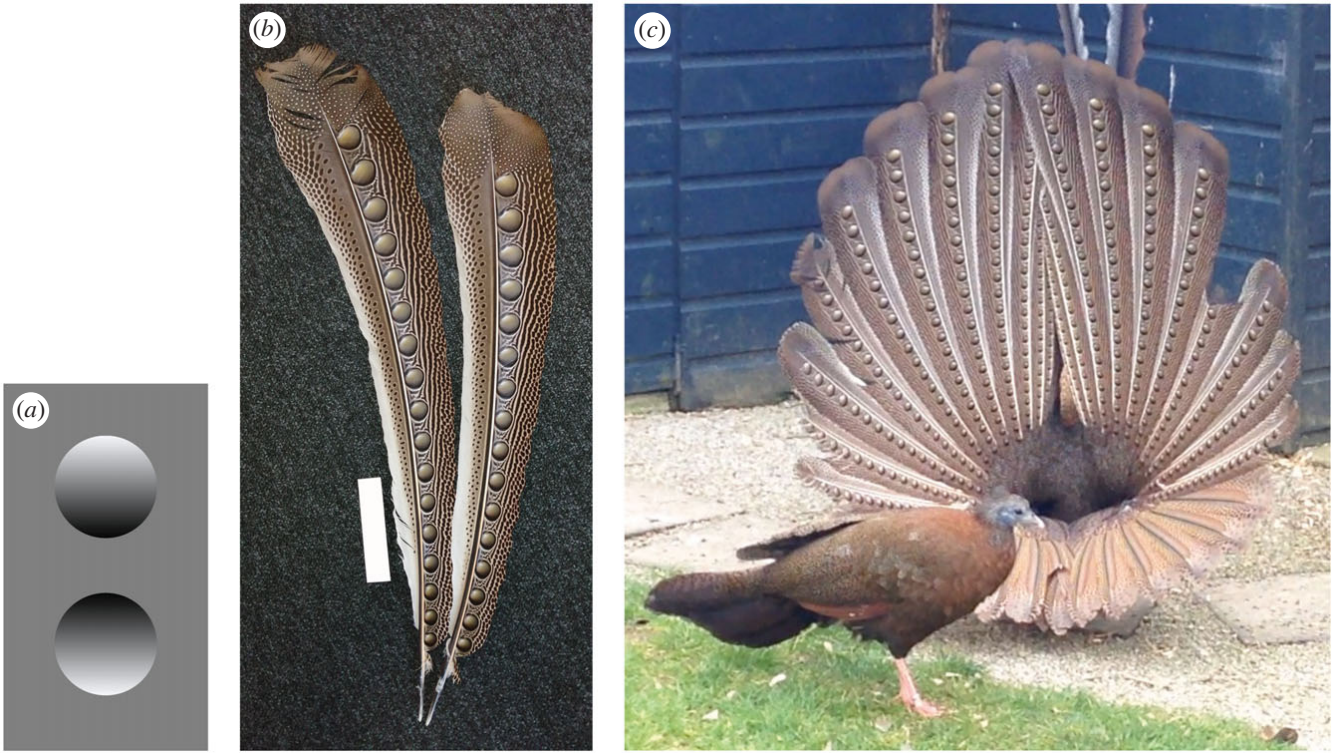
(*a*) Shading can create the appearance of 3D form on a flat surface, to appear convex (top) or concave (bottom); (*b*) feathers of the great argus showing the shaded ocelli, white scale bar shown is 10 cm; (*c*) a male great argus displaying to a female in front of him, where the male's head is tucked behind his wings (frame from video taken by David Woolcock).

Animals live in a three-dimensional (3D) world and use a variety of cues to resolve the location, size, orientation and shape of objects within their environment [1]. Objects reflecting light create shading (gradations of brightness across the object surface), specular highlights and cast shadows that contain information about shape. Human visual systems interpret shading cues based on the assumption that there is a single light source from above (figure 1*a*) [2], and imitating shading consistent with this assumption can create the impression of depth upon a flat surface, for example a 2D-shaded circle graded from a light top to a dark bottom is perceived as convex, and the inverse shading is perceived as concave (figure 1*a*) [2].

Other vertebrates can also resolve shape from shading patterns in the same way as humans ([3–6], but see [7]). Birds have been relatively well studied: pigeons, starlings and chickens can discriminate between images of convex and concave shapes based on shading patterns and shadows [4,5,8–10]. As a result, some animals such as caterpillars have evolved countershading (where the dorsal surface is darker than the ventral surface to counteract overhead illumination from the sun), which conceals 3D shading cues to enhance crypsis against predators [11]. The possibility that shape illusions could also be incorporated into sexual signals was first raised over 150 years ago by Charles Darwin and contemporaries [12,13]. The great argus *Argusianus argus* is a pheasant native to southeast Asia, and males have elongated primary feathers that have a single row of ocelli (eyespots) along each feather (figure 1*b*). These ocelli have a gradation of light to dark brown shading with a white region similar to a specular highlight on a sphere (figure 1*c*) [12,13]. However, it is unknown whether the putative viewer of this signal, conspecific females, perceives ocelli as 3D.

To test this, we used domesticated chickens *Gallus gallus domesticus* as a proxy for female great argus. Both are similarly sized ground feeding birds in the Phasianidae family, with domesticated chickens being descended from red jungefowl *Gallus gallus* and retaining comparable visual capabilities [14]. We first tested whether chickens could resolve shape from shading using surface shading cues alone, as shown previously [8]. We used a discrimination task to test whether chickens could discriminate 2D printed stimuli that had shading consistent with 3D convex or concave form [4,5]. We then presented the same birds with images of great argus ocelli presented as they appear during display (convex) and inverted (concave). We predicted that if birds perceive ocelli as 3D, individuals trained to convex shapes would select the ocellus in the natural position whereas those trained to concave shapes would select the inverted stimulus. If birds do not perceive them as 3D, they would select either stimulus randomly.

## 2. Methods

### (a) Stimuli

**Figure 2.**
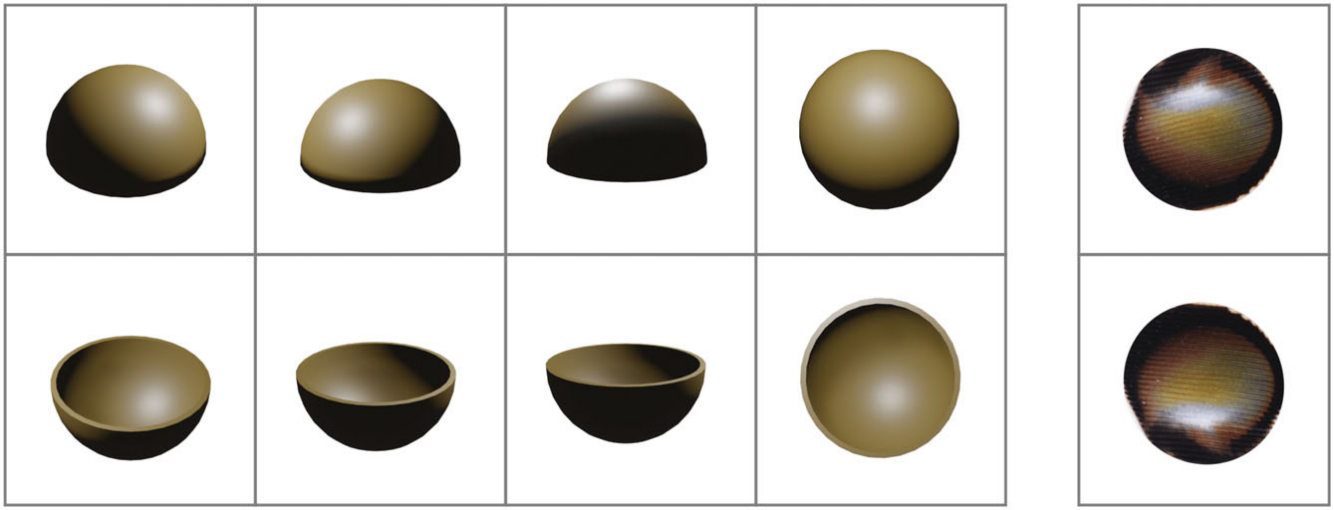
Examples of stimuli used in the 3D discrimination task and great argus test stimuli (far right column). Examples of convex stimuli are shown along the top row and concave along the bottom row (angle of viewing 37°, 23°, 16° and 90° (‘top down’ stimulus), respectively. The two stimuli used to test great argus ocelli on the far right were presented in a natural orientation (convex treatment, top) and rotated 180° (concave treatment, bottom).

We created 26 stimuli (13 concave and 13 convex) that differed in their lighting and shading to encourage the birds to attend to surface shape rather than using low-level visual processing or memorizing specific shading patterns [4,5]. Stimuli were created using the open-source software Blender (www.blender.org), where a hollow hemisphere was created and coloured brown (RGB 77, 62, 28). The hemisphere was exposed to an overhead light source at an angle of 55° to the vertical axis. The hemisphere was then rendered from three viewing angles (16°, 23° and 37° relative to horizontal plane) at 90° intervals around the horizontal plane, to create a total of 12 convex stimuli (figure 2). A ‘top down’ stimulus was created by rendering the hemisphere from an angle of 90° relative to the horizontal plane (full set shown in electronic supplementary material, figure S1). The hemisphere was then inverted on the horizontal plane and the rendering repeated from the same angles to create 13 equivalent concave stimuli (electronic supplementary material, figure S2). The stimuli were then resized to have a diameter of 23 mm and printed upon waterproof paper squares (35 mm × 35 mm; Xerox Premium NeverTear 120μ) so that all stimuli were presented upon a white background.

To create the great argus ocelli stimuli, we photographed eight wing feathers from male argus (sourced from Paradise Park, Cornwall and Edinburgh Zoo, Edinburgh). Feathers were lit from overhead by a full spectrum arc lamp (Iwasaki EyeColor 6500 K) and photographed using a Sony *α*7 fitted with a 28–70 mm lens. We randomly selected an ocellus from a randomly chosen feather and removed the feather background using the magic wand tool in ImageJ. The image was then resized to a diameter of 23 mm, and we printed two copies onto waterproof paper. One stimulus was rotated 180° to create the concave treatment (figure 2). We then imported the image into Inkscape and used the eyedropper tool to select a light brown on the ocellus, which was used as the base colour for the rendered hemispheres.

### (b) Experimental protocol

Domesticated chickens were sourced from local owners in Cornwall, UK. All birds were adult females from a range of breeds and had no reported issues with eyesight. Birds were visually inspected to confirm that they were generally in good health with no eye issues and no evidence of advanced senescence. All birds were visually isolated from flock mates during testing.

We created a two-choice chamber (60 × 30 × 30 cm, with a central divider creating two compartments of 30 × 30 cm) out of transparent Perspex with a grey Perspex back wall. The chamber had no floor or roof to encourage engagement. We stuck Velcro tabs in the middle of the back wall in each compartment at a height of 20 cm for presenting stimuli. We also attached a small tray (1 × 2 cm) made from a transparent acetate sheet (Card Crafts clear acetate 240μ) immediately underneath, so that food rewards were presented close to the stimulus. The front of the apparatus had a removable transparent Perspex screen to allow the birds to view both stimuli before making a choice.

Birds (*n* = 22) were randomly allocated to either the convex or concave treatment (*n* = 11 in each), where they were rewarded for selecting convex or concave stimuli, respectively. For each trial, the sample function in R [15] was used to randomly select a convex and a concave stimuli, and to randomly allocate the convex stimulus to the left or right compartment of the apparatus.

During trials, the experimenter stood behind the apparatus looking straight ahead to avoid providing visual cues. The stimuli were placed into the apparatus with the screen in place and the focal bird was given 20 s to inspect both stimuli. The screen was then removed and the time was recorded for the bird to make a choice by fully entering into one of the compartments. Response time was measured via peripheral vision which may have affected the precision of latency measures to a small degree. If 180 s passed without the bird entering a compartment, the trial was ended. If the bird correctly selected the correct stimulus, it was rewarded with a live mealworm (*Tenebrio molitor*) placed in the tray below the stimulus. Once the bird had made a choice, it was gently encouraged to leave the testing apparatus, the screen was replaced, and the next trial commenced. A bird was considered to discriminate between stimuli when it successfully chose the rewarded stimulus in six out of seven successive trials (86% correct choices).

Once a bird could discriminate between convex and concave stimuli, we presented the great argus stimuli using the same protocol. Each bird only received one presentation and was not rewarded for their choice.

### (c) Analysis

All analyses were run in R v.4.2 [15]. We used non-parametric tests when assumptions of parametric tests were not met. We tested whether latency to make a choice was correlated with accuracy (correct or not) using a Wilcoxon signed-rank test. To determine whether birds made decisions more quickly with repeated trials, we fit a linear mixed effects model using the *lme* function in *nlme* [16], with time as the response variable, trial number as a fixed effect and individual bird as a random effect. We also tested whether birds trained to convex or concave shapes differed in performance in terms of an initial preference for concave or convex shapes in the first trial, the number of trials needed to reach criterion and latency to choose, using a binomial test for initial preferences, a Kruskall–Wallis test for number of trials and a two-sample *t*-test for latency. We used a binomial test to determine whether birds performed above chance (50%) when presented with great argus stimuli.

## 3. Results

Of the 22 birds tested, two did not engage with the apparatus and were excluded from the study. The remaining 20 birds (*n* = 10 concave, *n* = 10 convex) all reached the learning criterion, needing an average of 10.6 ± 3.6 trials (mean ± s.d.) and a maximum of 19 trials. Out of a total of 205 trials, choices were made in all except for six trials. The average latency to make a choice was 34.0 ± 23.8 s (excluding the six trials where no choice was made), and there was no relationship between latency to choose and accuracy (*W* = 3593.5, *p* = 0.74). Individuals did not get faster at choosing during subsequent trials (*F*_1,184_ = 3.09, *p* = 0.08). There was also no difference between convex and concave treatments in terms of number of trials to reach criterion or latency to choose (number of trials: chi-squared = 0.013, *p* = 0.91; latency *t* = −1.08, d.f. = 18, *p* = 0.29), and no significant initial preference for either stimulus: 13 of 18 birds that made a choice in the first trial chose the convex stimulus (binomial test *p* = 0.096, 95%CI 0.47, 0.90). When presented with great argus stimuli, 17 of the 20 birds (85%) chose the correct stimulus (binomial test *p* = 0.003, 95%CI 0.62, 0.97) with a choice being made in an average of 31.9 ± 24.0 s (mean ± s.d.).

## 4. Discussion

We found that chickens could resolve shape from shading cues, and that they also likely interpret the shading patterns present on male great argus feathers as 3D. Despite Darwin suggesting over 150 years ago that these feathers had a 3D appearance, this study is the first test of whether birds also perceive them as 3D.

Our finding that chickens can resolve shape from shading is consistent with existing studies on chickens and other birds [4,5,8–10] and is perhaps unsurprising given that vision is considered their primary sense [17]. Birds learned the discrimination task in a relatively small number of trials [18–20]; however, some of our convex stimuli had a specular highlight in the upper half of the image and vice versa for concave stimuli, which birds could have used heuristically to discriminate between stimuli. We attempted to minimize this confound by including convex and concave stimuli with specular highlights in the central section of the shape, but future studies should include shapes illuminated from the side. Two of the 26 stimuli used during training were ‘top down’ stimuli which had similar shading patterns to the argus test stimuli, meaning some birds may have interpreted the test stimuli as previously seen training stimuli. However, birds had limited experience of these two stimuli during training, and most birds still responded to argus ocelli in the same way as artificial convex/concave spheres.

As Darwin surmised over 150 years ago, our results suggest that 3D illusions of shape can be created by animal patterning and used in courtship displays, although their role in signalling and female choice remains unclear. Ocelli without 3D effects are present in the courtship displays in two other genera within the Phasianidae (*Pavo* and *Polyplectron*), although the ocelli of Malayan peacock pheasants *Polyplectron malacense* appear slightly convex [21]. Phylogenetic relationships suggest that all three genera may have evolved ocelli independently [22], perhaps due to a female pre-existing bias for circular structures. Such a hypothetical bias could have originated from foraging behaviours, as spherical fruits contribute to great argus diet [23].

The apparent 3D form of the ocelli may enhance signal efficacy by increasing the overall salience of the display and holding the attention of viewing females [24]. Interestingly, we did not find that birds were inherently attracted to convex shapes as they did not choose this shape more often than by chance in the first trial; however, future work could investigate whether 3D form is inherently visually appealing. During display, the male fans out his feathers to create a conical shape and the decreasing size of the ocelli towards the base of the feathers (or ‘point’ of the cone) may create a tunnel effect for the viewing female through perspective cues [25]. This may further be enhanced by the movements of the male during display as he pulsates the cone of feathers towards the female. Modelling the female's perspective of the male's ocelli during display could allow us to determine whether such additional visual effects may be occurring [26]. Given that display movements, ocelli brightness and colour are factors in female choice in related species with similar displays [27–30], it seems likely that there are additional signal components involved in male argus courtship displays.

## Data Availability

The data are provided in the electronic supplementary material [31].
